# Influences on Perceived Feasibility of Animal-Based Measures in a Producer-Driven Welfare Benchmarking System

**DOI:** 10.3390/ani14182666

**Published:** 2024-09-13

**Authors:** Hannah Salvin, Jessica E. Monk, Linda M. Cafe, Steven Harden, Caroline Lee

**Affiliations:** 1 NSW Department of Primary Industries, Livestock Industries Centre, University of New England, Armidale, NSW 2351, Australia; hannah.ford@csiro.au (H.S.); linda.cafe@dpi.nsw.gov.au (L.M.C.); 2 CSIRO Agriculture and Food, FD McMaster Laboratory, Armidale, NSW 2350, Australia; jmonk5@une.edu.au; 3 School of Environmental and Rural Science, University of New England, Armidale, NSW 2351, Australia; 4 NSW Department of Primary Industries, Tamworth Agricultural Institute, Calala, NSW 2340, Australia; steven.harden@dpi.nsw.gov.au

**Keywords:** animal handling, quality of life, farmer, behaviour, welfare measures, stockpeople, feasibility, attitudes

## Abstract

**Simple Summary:**

Ongoing consumer support of the Australian red meat industry requires the industry to be transparent and accountable about the welfare of beef cattle under production. We conducted an online survey of Australian pasture-based beef cattle producers to determine the welfare measures they thought were important to include in a welfare benchmarking system and the feasibility of self-collecting animal-based data. Overall, the perceived feasibility of collecting animal-based data was related to land size and herd size, the producers’ overall attitude about the importance of quality of life in food-producing animals and the importance of individual cattle welfare measures to that producer. A well-designed and targeted programme to educate producers on why certain welfare measures are important will be crucial to increase uptake and retention in a voluntary welfare benchmarking system.

**Abstract:**

A voluntary, producer-driven welfare benchmarking system has been explored as a way of incentivising welfare improvement in pasture-based beef cattle and providing transparency and accountability to the industry. This study aimed to determine the acceptability and feasibility of measures for inclusion in a welfare benchmarking system and how this is influenced by respondents’ attitudes and beliefs. A survey was disseminated online to Australian producers in July 2020. Producers were asked to indicate the welfare measures (*n* = 59) they thought most important to check to determine if cattle on pasture-based farms have a good quality of life (QOL) and the feasibility of collecting animal-based welfare data and completing a stockperson attitudes questionnaire. Basic demographic and attitude data were also collected. Responses from 274 producers were included (52% male) with median land size 340 Ha (range 4–500,000) and herd size 200 head (2–200,000). Feasibility was related to QOL attitudes for 11 of the 17 animal-based measures (*p* < 0.01–0.02). Feasibility was also related to land or herd size but was not affected by other demographics, such as gender. In all significant dependencies, feasibility was reported as greater in those who thought it important to check the corresponding welfare measure. Producers who rated QOL as very important were also more likely to perceive the collection of animal-based data as feasible. A well-designed and targeted programme to educate producers on why certain welfare measures are important will be crucial to increase uptake and retention in a voluntary producer-driven welfare benchmarking scheme.

## 1. Introduction

There are a number of different factors, arguments and approaches that contribute towards the overall narrative on animal welfare within society, including, but not limited to, economics, religion, welfare science, political and animal rights arguments [1]. Each of these helps to develop and maintain the social licence under which livestock production operates, with an estimated AUD $3.9 billion downside risk to the Australian red meat industry if consumer support is lost [2]. Vital to the integrity of these arguments is access to transparent and accurate data on the welfare performance of livestock production enterprises. In Australia, a voluntary, producer-driven welfare benchmarking system has been explored as a way of incentivising the improvement of welfare in pasture-based beef cattle and providing transparency and accountability to the industry [3,4].

A primary concern when determining suitable measures for inclusion in any welfare assessment system is their reliability, validity and feasibility [4]. Our ethical approach to animals, both as a society, but more importantly, as individuals, will influence perceptions of the moral status of animals and therefore how they should be treated [5]. Importantly, stakeholder perceptions of what constitutes animal welfare, as well as those of the animal science community, need to be considered when developing assessment or assurance systems, as they can often differ [6,7,8]. Selecting measures of animal welfare that are both scientifically robust and widely accepted as valid by a diverse range of stakeholders is also vital if on-farm welfare assessment is to be incorporated into local or national regulations [9].

Demographics and beliefs have been shown to affect attitudes towards animal welfare. A study of veterinary students by Colombo et al. [10] found that females showed a higher level of empathy towards animals than their male counterparts. Similarly, the demographics of livestock producers, such as farming sector and gender, influenced how participants judged the welfare of animals and the level of importance given to health and natural behaviours when assessing positive welfare [11]. Even within the category of producers, differences in attitudes have been demonstrated based on alignment with different assurance schemes. Pig producers who participated in organic or animal-welfare-related assurance schemes placed a higher importance on the ability of animals to display natural behaviours, while producers in product quality assurance schemes were more concerned with animal health and productivity [12]. Stakeholders across varied demographics and beliefs should be consulted to develop a welfare assurance system that is accepted by the broader society.

Although completely satisfying all stakeholders is unrealistic, it is critical to engage with a wide range of producers to understand their views and beliefs on animal welfare and take these into consideration when selecting measures for inclusion in a producer-driven welfare benchmarking system. This aligns with Icek Ajzen’s theory of planned behaviour, where an intention to do something is a precondition to undertaking the behaviour, and the intention is determined by the person’s attitude towards the behaviour, their perceived control over the behaviour and the opinions of respected others. Importantly, the attitudes need to directly relate to the behaviour and not be more general in nature to best link behaviour and attitudes together [13]. Developing welfare measures that producers perceive as useful and in keeping with their values will make them more likely to accept and implement them [14,15,16].

The purpose of this study was to determine the relative importance of a suite of welfare measures proposed for inclusion in a welfare benchmarking system aimed at Australian pasture-based or extensive beef cattle producers. For the measures for which animal-based data would need to be collected by producers, feasibility was also assessed, and its link to demographics, beliefs and the perceived importance of the different welfare measures was explored. Ensuring that any welfare assessment scheme is valid, fit for purpose and in line with producer values and beliefs is important for increasing its success if rolled out voluntarily or as part of updated animal welfare regulations. Understanding the factors that may affect the perceived feasibility of data collection is also important for tailoring education and training systems to best target areas where uptake may be lower.

## 2. Materials and Methods

A survey was devised to inform the development of a system to assess and benchmark welfare in beef cattle (available as Supplementary Materials). The survey and any promotional material used to disseminate the survey was approved for use by the CSIRO Social Science Human Research Ethics Committee, approval # 067/20. The survey was open to all Australian residents aged 18 years or older for 4 weeks from 26 June to 26 July 2020. A rural producer audience was targeted to ensure there was a strong representation of respondents with experience owning or working on a pasture-based beef property. Responses from non-producers, i.e., those who had NOT owned or worked on a pasture-based beef cattle property in the last 10 years, were used elsewhere in the development of the benchmarking project, and their data are not presented here. A referral sampling technique was used, in which the survey was disseminated to key industry stakeholders, who were asked to further disseminate the survey to their members or followers. The industry stakeholders provided with a link to the survey and asked to forward it to their members included all Australian beef breed societies (*n* = 21) [17], RSPCA Australia, the Southern, Northern and Western Australian Research Councils and Meat and Livestock Australia. A link to the survey was also posted a single time on the social media accounts (Twitter and Facebook) of NSW DPI, CSIRO and Meat and Livestock Australia. An advantage of this technique is that it has the potential to reach a larger population of suitable participants than could be accessed by the researcher’s network alone. The number of stakeholders who forwarded the details of the survey, their membership base and the view rates for social media are not known; it is therefore impossible to know the full reach of the survey and to calculate a response rate.

The survey was divided into two parts, aiming to (1) determine which measures of animal welfare are most important to assess and (2) to determine the feasibility of collecting animal-based data, as perceived by beef producers.

In part one of the survey, participants were presented with the following vignette:

*“Imagine you have been asked to inspect a pasture-based beef farm and to decide if you think the cattle there have a good quality of life. What do you think would be the MOST important things to check on that farm to prove that the cattle have a good quality of life? Try to consider what you would most want to know about regardless of whether you think it would be easy or practical to measure”.*

Participants could then freely select (tick) as many of the listed welfare measures (*n* = 59) as they thought should be checked to prove that cattle have a good quality of life. The welfare measures presented in the survey as being important to assess were derived from 1 or more of the 150 indicators identified during a review of existing cattle welfare assurance schemes (Global Animal Partnership, Bord Bia, Red tractor, Welfarecheck, AssureWel, Welfare Quality, UC Davis cow/calf and Bio Austria), the scientific literature [18] and the Australian Animal Welfare Standards and Guidelines for Cattle [19]. Measures were categorised into the following sections: health (*n* = 10), farm environment (*n* = 6), food and water (*n* = 12), farm management (*n* = 7), handling (*n* = 12), behaviour of cattle and stockpeople (*n* = 7) and husbandry (*n* = 5). An additional statement of “None of the above needs to be checked” was added to the end of each section, giving a total of 66 options that could be selected. Details of the individual measures are presented in the results.

The term “quality of life” was used instead of “welfare”, as it was thought to be more broadly understood and to not carry negative connotations. Quality of life can also be considered as finding a balance of positives over negatives and aligns with the concept of welfare as a continuum that can be benchmarked [20].

At the end of part one, participants were asked to complete basic demographic questions, including, age, gender and postcode. Questions were also asked relating to their beliefs and understanding of beef production, including beef consumption status, the importance of quality of life for animals used for food production, the level of interaction with pasture-based beef farms in the past 10 years, self-reported knowledge of pasture-based beef production systems and estimation of the overall quality of life currently experienced by Australian beef cattle in pasture-based systems. Pasture-based systems were defined as those in which cattle are grazed in open paddocks on a predominantly pasture diet.

Participants who indicated that they had owned or worked on a pasture-based beef cattle property in the last 10 years were directed to part two of the survey. Part two contained further demographic questions about the properties on which respondents had worked, including the size of the property, total head of cattle and factors that would encourage them to use a welfare benchmarking tool. They were then asked to indicate how feasible it would be for them to collect and record animal-based welfare data, including body condition score, weight, health conditions, calving difficulties, signs of heat stress and exhaustion during mustering and herd demeanour. Responses were recorded on a 5-point Likert scale, with response options including “I already collect it”, “Highly feasible”, “Feasible”, “Unfeasible” and “Highly unfeasible”. If respondents indicated that data collection was unfeasible, they were asked to select one or more reasons from “Time required”, “Additional personnel required”, “Causes additional stress to the cattle” and/or “Other”. Additional question-specific reasons for data collection being unfeasible were also offered for some questions, e.g., “I don’t have access to weigh scales (weight)” or “I don’t check them during calving” (calving difficulty).

For handling data collected during mustering and yarding, participants were asked to indicate how confident they were to record animal-based data either during or after mustering or handling cattle in the yards. The data for mustering included how often the dogs bit cattle, the average speed cattle travelled at and how often cattle broke away from the herd. The data for yarding included the percentage of cattle requiring force to move, trips and falls, getting stuck, trying to escape, mis-catches in the head bail, vocalising before procedures are performed and the number of yard locations where flow is inhibited. The response options were “Confident to record accurately”, “Confident to record an estimate”, “It’s too hard to see the cattle during mustering/yarding to record this”, “It would take too long to record this”, “It’s too hard to keep track of the details during mustering/yarding” and “Other”. Participants were able to select multiple options. The question relating to dogs biting cattle during mustering also included an additional response option of “I don’t use dogs to muster”.

Participants were also asked to indicate if they would have any concerns completing a stockperson attitudes questionnaire or asking anyone else handling cattle to complete one as a potential proxy measure of handling quality. The response options included “Yes”, “No”, “I am not involved in handling cattle” or “No one else handles the cattle”.

All statistics were completed using R 4.2.0 statistical analysis software [21]. The participants’ demographics data were summarised using basic summary statistics and graphs. Postcodes were used to group participants into the remoteness categories of “Major city”, “Regional” or “Remote”. These categories were determined according to the Australian Standard Geographical Classification (ASGC) Remoteness Structure [22]. Property sizes (<100 ha, 101–1000 ha, 1001–10,000 ha, >10,000 ha) and herd sizes (<100, 101–300, 301–1000, >1000) were grouped into four categories. For analysis, states were grouped into New South Wales and Australian Capital Territory (NSW/ACT), Northern Territory, South Australia and Western Australia (NT/SA/WA), Queensland (QLD) and Tasmania and Victoria (TAS/VIC) to create more even-sized groups.

For each of the welfare aspects listed in part one of the survey, the total number of participants who deemed each measure to be important was tallied and graphed.

The feasibility responses were condensed into Feasible (“I already collect it”, “Highly feasible” and “Feasible”) and Unfeasible (“Unfeasible” and “Highly unfeasible”). The responses on the confidence to record were condensed into “Confident to record accurately”, “Confident to record an estimate only” and “Unfeasible” (“It’s too hard to see”, “It would take too long” and “It’s too hard to keep track”). The responses of “Other” only or “I don’t use dogs to muster” were removed from subsequent analyses, as they did not provide information on confidence levels.

The relationships between demographics or beliefs and understanding, and perceived feasibility of animal-based measures were assessed by generating contingency tables and using a Pearson chi-squared test of independence to determine whether the association was significant. Frequencies in the contingency table were also modelled by fitting generalised linear models with a Poisson distribution, including both questions and their interactions, to determine estimated group percentages and the standard error. The step function in R [21] was used to determine the preferred or final model based on Akaike’s information criteria. The scope option was used, so that the main terms were always retained, while the interaction terms could be omitted.

Similarly, the relationships between the feasibility of collecting animal-based data in part two and the likeliness to consider a welfare measure important to assess in part one were tested for independence and modelled for those combinations for which there was a probable link. The combinations for which the relationships were modelled are presented in Table 1.

## 3. Results

### 3.1. Demographics and Beliefs

**Figure 1:**
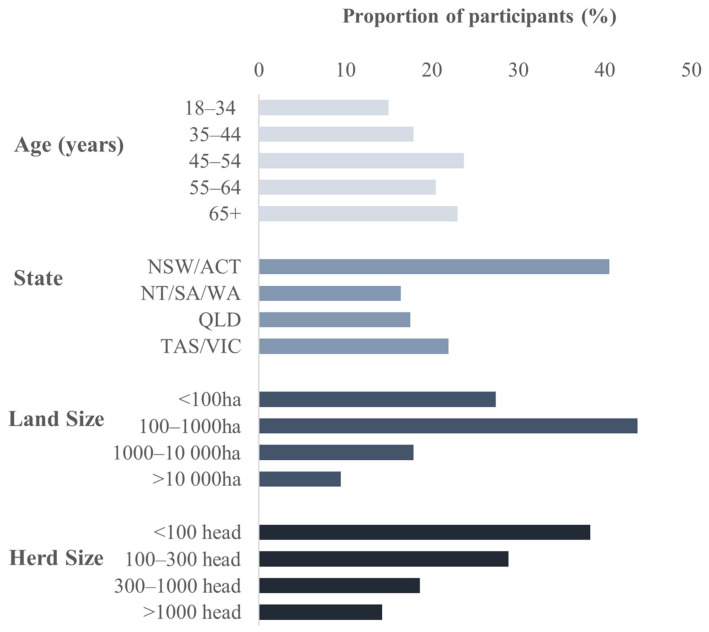
Demographic spread of survey respondents in groupings by age, state of residence and the land size and herd size of the pasture-based beef cattle property they had owned or worked on in the last ten years.

A total of 274 producers who had owned or worked on a beef cattle property completed at least part of the survey and were included in the analysis. The gender of participants was relatively evenly distributed, with 47.8% females, 51.5% males and 0.7% who did not indicate a gender. Of the participants, 97.4% ate beef products, 2.2% purchased beef products for their households but did not eat beef, and 0.4% did not consume or purchase beef products. Most participants were from regional areas (82.1%), with a small number from major cities (6.9%) or remote (7.2%) areas. The median land size of the beef property participants had owned or worked on in the last ten years was 340 hectares (average: 19,880 Ha; range: 4–700,000 Ha), with a median herd size of 200 head (range: 2–200,000). The distributions of age, state, land size and herd size groups used for analysis are shown in Figure 1.

Most participants reported their knowledge of pasture-based beef cattle production systems as excellent or good (94.2%), with few reporting their knowledge as fair (5.1%) or poor/non-existent (0.4%). The importance of animals raised for food production in Australia having a good quality of life was reported as very important by 74.8% of participants and important or moderately important by 25.2% of participants. No participants felt that quality of life was only slightly important, or not at all. The overall quality of life experienced by beef cattle living in Australian pasture-based systems was thought to be very good or good by 81% of participants, acceptable by 13.8% and poor or very poor by 2.9% of participants. A small number (1.8%) of participants indicated that they did not know the overall quality of life experienced by beef cattle in Australia.

### 3.2. Feasibility of Animal-Based Welfare Measures

**Figure 2:**
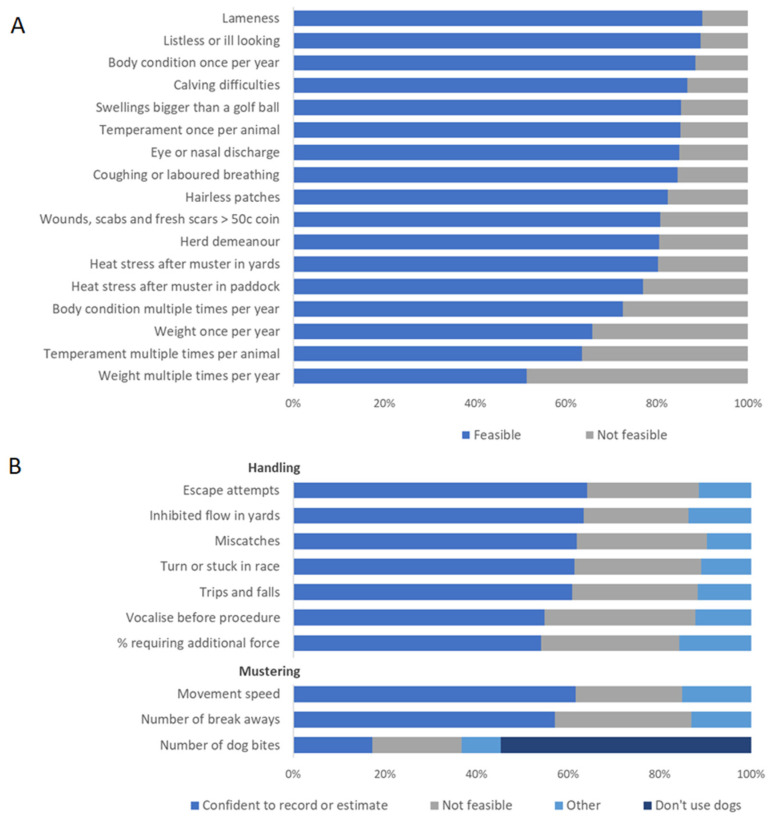
(**A**) Proportion of respondents who considered each of the listed measures to be feasible (“I already collect it”, “Highly feasible” or “Feasible”) or not feasible (“Unfeasible” or “Highly unfeasible”) to collect and record on farm and (**B**) proportion of respondents who were confident to record or estimate each of the listed measures, who thought it was not feasible or who selected “Other” (free text).

In part two of the survey, over 80% of respondents indicated that it would be feasible for them to record health issues at yarding events, body condition scores at least once per year, temperament scores at least once per animal, herd demeanour in the first 5 min after mustering to yards, heat stress after mustering to yards and the number of cows with calving difficulties (Figure 2A). Recording body weight multiple times each year was considered to be unfeasible for approximately 50% of respondents. Of the 130 respondents who indicated this would not be feasible, 55 (42%) indicated it was because they did not have access to weigh scales.

Animal-based data on mustering and handling quality were generally considered to be less feasible to record (Figure 2B). Between 55 and 65% of respondents indicated they could record handling data either accurately or as an estimate. The “Time required” followed by the “Additional personnel required” for data collection were the reasons given for data not being feasible to collect. Within the “Other” category, the comments made were related to the constantly changing nature of the data over time, a tendency to be always assessing these behaviours but not formally scoring or recoding the data, subjectivity of the data, logistics on large properties, potential to be distracted by scoring during handling and concern over excessive paperwork or red tape.

The proportions of respondents who indicated they would complete a stockperson attitudes questionnaire or enlist others who handle cattle to complete one were 93% and 86%, respectively. For those who said they would not, the reasons for concern included that producers should be trusted to care for their animals, fear of offending staff or family, it would be easy to guess the desired answers and that this is something managers could judge themselves. Whether respondents did or did not have concerns about completing a questionnaire was not related to any demographic or belief responses.

### 3.3. Relationship between Demographics and Perceived Feasibility of Measures

There were few to none significant relationships between demographics (age, gender, state, beef consumption or self-reported knowledge of pasture-based beef production systems) and the feasibility of collecting animal-based data or the confidence of participants recording data. However, the importance of animals raised for food production having a good quality of life, land size and herd size demographics had a strong relationship with feasibility and the confidence to record data. Remoteness and overall quality of life experienced by pasture-based beef cattle in Australia had a moderate relationship with feasibility but are not reported in detail here due to their similarity in terms of effect size and direction to the land size and importance of quality of life results, respectively. Overall quality of life experienced by Australian cattle is also highly subjective and may not be truly reflective of the current situation.

The relationships between feasibility of animal-based data collection and the importance of animals raised for food production having a good quality of life, land size and herd size groupings are shown in Table 2. In all significant relationships between the importance of a good quality of life and feasibility of data collection, feasibility increased as importance increased. Of the respondents who thought quality of life was very important, 77–84% reported animal-based data as feasible to collect, while 48–68% said it was unfeasible. Conversely, of those who thought quality of life was only important or moderately important, 16–23% reported data collection as feasible and 32–52% as unfeasible.

In all significant relationships between land size and feasibility, feasibility decreased as land size increased beyond 1000 Ha. For example, 28–49% of respondents with land sizes < 1000 Ha reported animal-based data collection as feasible compared with only 4–9% of respondents with land sizes > 10,000 Ha. Similarly, in all significant relationships between herd size and feasibility, feasibility decreased as herd size increased. The exception was an increased proportion of respondents with herd sizes < 100 head who considered weighing cattle unfeasible, driven by a lack of access to weigh scales.

The relationships between confidence to record handling data and the importance of animals raised for food production having a good quality of life and land size are shown in Table 3. There were no significant relationships between herd size and confidence to record any handling data during mustering or yarding. In all significant relationships between the importance of a good quality of life and confidence to record, confidence increased as importance increased. Of the respondents who thought quality of life was very important, 83–91% reported they could accurately record animal-based data, while 59–70% said it was unfeasible. Conversely, of those who thought quality of life was only important or moderately important, 9–17% reported they could accurately record animal-based data, and 30–41% said it was unfeasible. In all significant relationships between land size and confidence to record handling data, confidence decreased as land size increased beyond 1000 Ha.

### 3.4. Importance of Welfare Measures

**Figure 3:**
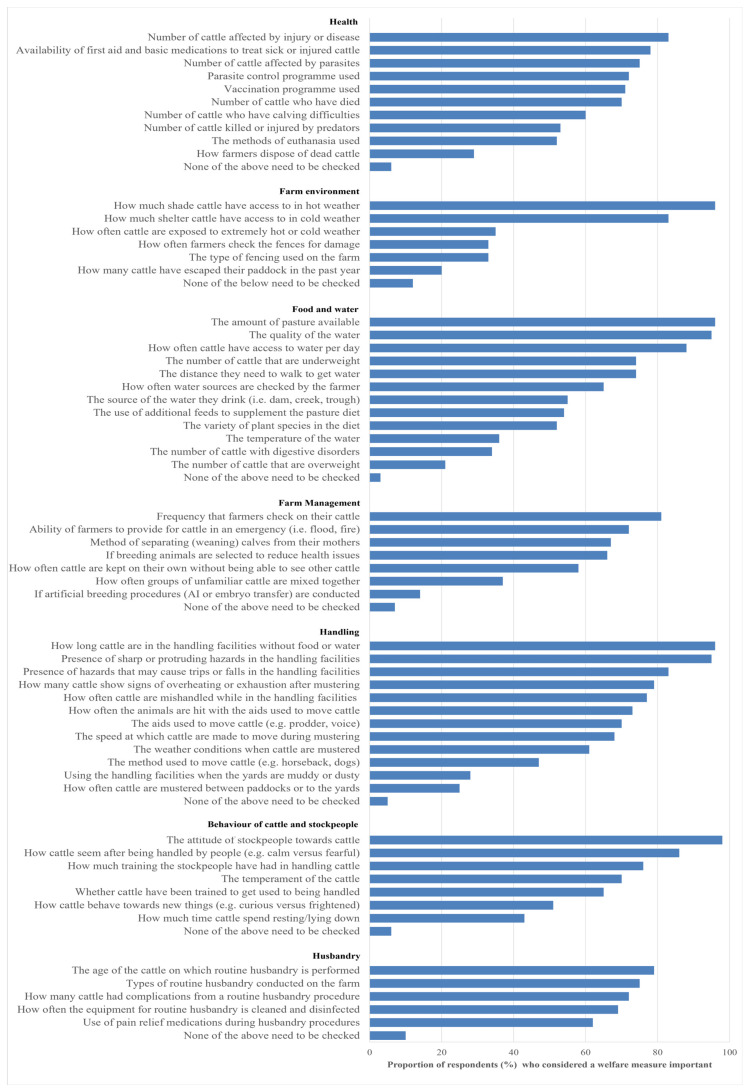
Proportion of respondents (%) who indicated that the given welfare measures should be checked to determine the quality of life of beef cattle in pasture-based systems.

The proportion of respondents who indicated each welfare measure as important to check to prove that cattle have a good quality of life in pasture-based systems is shown in Figure 3. Within subcategories, welfare measures are sorted from the highest to the lowest proportion of respondents. Access to shade, availability of pasture, water quality, how long animals are kept in the yards, the presence of hazards in the yards and the attitudes of stockpeople were indicated as important by over 90% of respondents. Conversely, how farmers dispose of dead cattle, cattle escapes, overweight cattle, use of artificial breeding, using the yards when muddy or dusty and the number of times cattle are mustered were deemed important by less than 30% of respondents.

### 3.5. Relationship between Importance and Perceived Feasibility

Where multiple comparisons are listed, the measures assessed for importance are indicated by A: B: C: …, while on-animal data collection measures assessed for feasibility or confidence to record are indicated by 1: 2: 3: …

#### 3.5.1. Health and Body Condition

There was a strong relationship between the feasibility of data collection for health conditions and the percentage of respondents who thought that various health-related measures were important to check to determine the quality of life (Table 4). In all significant relationships, the proportion of respondents who reported data collection as feasible was higher in those who also reported that health measures were important to check.

There was no relationship between the importance of checking A: the frequency of farmers checking on their cattle or B: the number of cattle with calving difficulty and the feasibility of checking for calving difficulties.

The importance of checking the number of cattle that were over- or underweight was related to the feasibility of 1: assessing body condition score once (*p* < 0.01–0.01) or multiple times (*p* < 0.01–0.01) and 2: weighing cattle once per year (*p* = 0.01–0.04) but not weighing cattle multiple times (*p* = 0.1–0.21). For checking the number of animals that are over- or underweight, those who reported it as important were more likely to also report it was feasible to assess body condition or weight.

There was no relationship between the importance of checking the use of supplementary feeds (*p* = 0.29–0.83) and the feasibility of 1: assessing body condition or 2: weight.

#### 3.5.2. Mustering and Handling

There were a number of significant relationships between the importance of measures and the feasibility of animal-based data collection during mustering and handling. For all significant mustering and handling measures, those who reported they were important were more likely to also report it was feasible to collect data. Similarly, the proportion of those who reported the aspects as important increased as the confidence to collect data increased.

There was a relationship between the importance of checking A: the number of cattle showing signs of overheating or exhaustion during mustering or B: the weather conditions during mustering and the feasibility of collecting data on the number of cattle displaying heat stress after 1: mustering to a new paddock (*p* < 0.01) or 2: to the yards (*p* < 0.01).

The importance of checking A: the speed at which cattle are made to move during mustering; B: the method used to muster cattle (e.g., dogs, motorbike, etc.); or C: the type and use of handling aids showed a strong relationship with respondents’ confidence in collecting animal-based data during mustering, including 1: how often dogs bit cattle (*p* < 0.01); 2: the average speed cattle travelled at (walk, trot, run; *p* < 0.01–0.01); and 3: how often individuals or groups of cattle broke away from the larger herd (*p* < 0.01).

There were significant relationships between the importance of checking for A: the presence of sharp or protruding hazards in the yards or B: the presence of hazards that may make the animal trip or fall in the yards and the confidence of respondents in collecting most of the animal-based data during handling (see Table 1 for a description of the measures). However, the importance of checking for the presence of hazards that may cause trips or falls in the yards was not related to the confidence in assessing the percentage of cattle that require additional force to move (*p* = 0.13). Similarly, the importance of checking for the presence of sharp or protruding hazards in the yards was not related to the confidence in assessing 1: the percentage of cattle who trip or fall (*p* = 0.07); 2: those who get stuck in the race or crush (*p* = 0.1); or 3: those who vocalise prior to a procedure (*p* = 0.37).

The reported importance of checking A: how often the cattle are mishandled during handling or B: the type and use of handling aids was strongly related to the confidence of respondents in collecting all animal-based data during handling (*p* < 0.01–0.01).

#### 3.5.3. Behaviour of Cattle and Stockpeople

The reported importance of checking A: how cattle appear after being handled (*p* < 0.01) or B: how cattle behave towards new things (*p* < 0.01) was related to the feasibility of assessing how the herd appears (herd demeanour) after interacting with people using a herd-based qualitative behavioural assessment.

The importance of checking the temperament of cattle (*p* = 0.42–0.7) and the feasibility of collecting temperament data using 1: the crush score (1–5) or 2: the crush exit score (walk, trot, run or jump) were not related. However, there was a relationship between the importance of how cattle behave towards new things and the feasibility of collecting temperament data once (*p* = 0.03) but not multiple times per year (*p* = 0.08) while on the property.

There was a significant relationship between the reported importance of checking how much training stockpeople have had and the concern respondents had over 1: enlisting others to complete a stockperson attitudes questionnaire (*p* < 0.01) but not 2: completing one themselves (*p* = 0.07). Conversely, there was a relationship between the importance of checking the attitudes of stockpeople towards cattle and concern among respondents over 1: completing a stockperson attitudes questionnaire (*p* < 0.01) but not 2: asking others to complete one (*p* = 0.08). Again, for all significant relationships, the proportion of respondents who reported a measure as important was higher for those who reported it as feasible or who did not have concerns completing or asking others to complete a stockperson attitudes questionnaire.

## 4. Discussion

The importance of considering public perceptions as well as scientific knowledge when developing welfare assessment schemes has been previously investigated. For example, during the development of the well-utilised European system Welfare Quality^®^, care was taken to incorporate the societal opinions of both producers and community members [6]. Here, we looked at not only what welfare measures are important but also the feasibility of collecting animal-based data and the interplay between feasibility, attitudes and demographics. A better understanding of this interplay will facilitate a welfare benchmarking system that is valid but also fit for purpose and acceptable to the producers who must adopt it.

The feasibility of collecting health, body condition and temperament animal-based measures was generally reported as high. Feasibility decreased when respondents were asked to collect measures multiple times per year, if land size exceeded 1000 Ha and for increasing herd sizes. Feasibility was reported as higher if the importance placed on the quality of life of animals produced for food was also reported as high, particularly for the measures collected multiple times. Females were overrepresented among the respondents (48%) compared to the national census [23], where 22–42% of beef cattle employees in Australia were reported to be female. Regardless, there was little to no relationship between gender and the reported feasibility of collecting animal-based measures or any other demographic factors. For the demographics of beef consumption and self-reported knowledge of pasture-based beef cattle production systems, the proportion of respondents who did not consume beef and who reported having fair or poor/non-existent knowledge, respectively, was very low and likely precluded the ability to determine any differences between these demographic groups.

It was surprising that gender did not affect feasibility, given the links found between feasibility and the reported importance of quality of life, and considering past research showing gender differences in empathy for animals [10] and how it is assessed [11]. This could be due to a response bias towards those producers who already have an active interest in welfare, with no participants reporting that quality of life in food-producing animals was not or was only slightly important. Alternatively, it could be due to the loose nature of the link between the importance of quality of life in all food-producing animals and the feasibility of collecting specific animal-based measures, as suggested by Ajzen’s theory of planned behaviour [13]. Kauppinen et al. [24] similarly found no difference in attitudes towards animal welfare between men and women and posited that producers may be more homogenous in their attitudes than other demographic groups.

Australia is unique compared to many other countries in terms of the size and scope of its beef production. In the European Union (EU), over 60% of all farms (both cropping and livestock) are less than 5 Ha, with only 7.5% of all EU farms greater than 50 Ha in size [25]—a key reason why many European-based assurance schemes are not fit for purpose in the Australian landscape. At the time of this survey, the average area operated by beef cattle producers in Australia was 14,870 Ha [26]. Among the respondents in this survey, the average land size was 19,880 Ha, slightly more than the national average, but likely still representative. It was not unexpected that this study found a number of significant relationships between the feasibility of collecting animal-based measures and land or herd size, given the logistics of collecting data over large areas or for large numbers of cattle. The confidence to record measures during mustering and yarding was conversely not related to herd size. This may be because, regardless of the overall number of head on the property, mobs of cattle are generally brought into the yards in smaller groups suited to the size of the holding facilities available.

Confidence in collecting data during mustering and yarding was lower. This is understandable, given the logistics of recording data while mustering, especially if conducted on horseback or on a motorbike, and a likely shortage of spare stockpeople in the yards to record data. In Australia, nearly three-quarters of employees in beef operations are owner-operators or unpaid family [23]. Stockperson attitudes questionnaires have been shown to provide a suitable proxy measure of handling quality both in other species and in cattle [27,28,29]. Here, the attitude of stockpeople towards cattle was reported as important by the highest number of respondents across all measures. This is in keeping with a survey of 361 people involved with the cattle industry by Phillips et al. [30], who found that the most important practice affecting the welfare of Australian beef cattle was stockmanship. The feasibility of collecting attitude data was also high, with 93% of respondents reporting not having any concerns completing a stockperson attitudes questionnaire themselves, although this decreased to 86% when asking others to complete one. This could be due to the sensitive nature of asking employees or family members about underlying attitudes and personality traits such as sympathy and would need to be carefully managed to ensure the anonymity of responses. The use of a stockperson attitudes questionnaire to assess handling quality within a welfare benchmarking system warrants further investigation.

Overwhelmingly, across all significant relationships between the importance of checking an aspect to prove animals have a good quality of life and the feasibility of collecting animal-based measures relating to that aspect, feasibility was higher in those who thought the aspect was important. This contrasts with the research of Kauppinen et al. [31], who found that in Finnish pig and dairy cattle producers, the intention to provide good animal welfare was explained by their underlying attitudes, but the perceived behavioural control, i.e., how easy it would be to implement a welfare-improving measure, was not directly connected to the intention. The strength and consistency of the relationship in this study, however, suggest that feasibility is not a fixed construct relating only to the logistics of herd or property size but can be influenced by a person’s beliefs and attitudes. As people’s behaviour and attitudes towards livestock have been shown to be favourably improved through training [32,33], the perceived feasibility of collecting animal-based data in a producer-driven welfare benchmarking system could also be improved through education programmes. An appropriately designed industry education programme will be vital for increasing the awareness, uptake and success of a welfare benchmarking system.

## 5. Conclusions

Welfare assessment systems must be designed to be reliable, valid and feasible. To achieve this, it is important that producer and community opinions are sought, as well as drawing from the scientific knowledge base. Here, we identified the most important welfare measures that producer respondents thought should be assessed, as well as determining the feasibility of collecting animal-based data across a range of production system sizes and locations. We identified that demographic factors such as land size and herd size affect the feasibility of collecting data, particularly on properties over 1000 Ha. Importantly, we found that feasibility was in many cases related to the respondent’s attitude towards welfare in food-producing animals in general and towards several individual welfare measures in particular. Specifically, producers who thought it was very important that food-producing animals have a good quality of life were generally more likely to perceive the collection of animal-based data as feasible. A well-designed and targeted programme to educate producers on why certain welfare measures are important will be crucial to increase uptake and retention in a voluntary producer-driven welfare benchmarking scheme.

## Figures and Tables

**Table 1:** Combinations (X) for which the relationships between (A) the importance of assessing welfare measures (part one) and (B) the feasibility of data collection (part two) were evaluated, based on a priori expected relationships.

A: Measures Assessed for Importance	B: Feasibility of Data Collection									
	Health Issues ^1^	Calving Difficulties	Body Condition Score	Weight ^2^	Handling Quality—Yarding ^3^	Handling Quality—Mustering ^4^	Heat Stress	Demeanour	Temperament ^5^	Stockperson Attitudes Questionnaire
No. affected by injury/disease	X									
No. affected by parasites	X									
Vaccination programme	X									
Parasite control programme	X									
Availability of first aid/medication	X									
Frequency of checking cattle	X	X								
No. of calving difficulties		X								
No. of cattle underweight			X	X						
No. of cattle overweight			X	X						
Supplementary feeding			X	X						
Hazards in yards					X					
Type of handling aids					X					
Frequency hit with aids					X					
Frequency of mishandling					X					
Mustering speed						X				
Mustering method						X				
No. of cattle with heat stress							X			
Weather during mustering							X			
Cattle response to handling								X		
Cattle response to novelty								X	X	
Cattle temperament									X	
Stockpeople training										X
Stockpeople attitude										X

^1^. Health issues: lameness; listless or ill-looking; swellings bigger than a golf ball; eye or nasal discharge; coughing or laboured breathing; hairless patches; wounds, scabs and fresh scars > AUD 50c piece. ^2^. Weight measured once or multiple times a year. ^3^. Measures of handling quality during yarding: percentage of cattle requiring additional force to move; how many cattle trip or fall in the yards/race/crush; how many cattle turn around or get stuck in the race/crush; how many cattle are mis-caught in the head bail of the crush; how many cattle try to escape; how many cattle vocalise prior to having any procedure performed; number of locations in the yards where cattle flow is regularly inhibited. ^4^. Measures of handling quality during mustering: how often dogs bit cattle; average speed cattle travelled at; how often individuals or groups of cattle broke away from control. ^5^. Temperament: crush score (1–5); crush exit score (walk, trot, run or jump).

**Table 2:** Relationship between feasibility of collecting animal-based data and (A) the importance of animals raised for food production in Australia having good quality of life (QOL), (B) land size and (C) herd size. Significant *p*-values (<0.05, highlighted), as indicated by Pearson’s chi-square test of independence, suggest that the two responses are related.

	(A) Importance of QOL	(B) Land Size	(C) Herd Size
**Body condition score**			
Multiple/year	<0.01	<0.01	<0.01
Once	0.06	0.06	0.06
**Body weight**			
Multiple/year	0.01	0.02	<0.01
Once	0.62	0.42	<0.01
**Temperament**			
Multiple while on property	<0.01	<0.01	<0.01
Once	0.19	<0.01	<0.01
**Health conditions**			
Lameness	0.08	0.01	0.02
Hairless patches	<0.01	<0.01	<0.01
Open wounds	<0.01	<0.01	<0.01
Swellings	<0.01	0.01	0.02
Eye/nose discharge	0.01	<0.01	<0.01
Coughing	0.02	<0.01	0.01
Listlessness	0.02	<0.01	<0.01
**Calving difficultly**	<0.01	<0.01	<0.01
**Heat stress**			
Muster to new paddock	0.02	<0.01	<0.01
Muster to yards	0.11	<0.01	<0.01
**Demeanour**	0.06	0.01	0.19

**Table 3:** Relationship between confidence to record animal-based data during mustering and yarding and (A) the importance of animals raised for food production in Australia having good quality of life (QOL) and (B) land size. Significant *p*-values (<0.05, highlighted), as indicated by Pearson’s chi-square test of independence, suggest that the two responses are related.

	(A) Importance of QOL	(B) Land Size
**Mustering**		
How often dogs bit cattle	0.01	0.01
Average speed cattle travelled at	<0.01	<0.01
How often cattle broke away from herd	<0.01	<0.01
**Handling**		
Percentage of cattle requiring force to move	0.01	0.08
How many cattle trip/fall	0.21	0.01
How many cattle get stuck in race/crush	0.15	0.08
How many cattle are mis-caught in head bail	0.05	0.01
How many cattle try to escape	0.01	0.19
How many cattle vocalise before procedure	0.02	0.02
Yard locations where flow is inhibited	0.04	0.04

**Table 4:** Relationship between feasibility of collecting animal-based data during mustering and yarding and the importance of checking various health-related measures when determining quality of life. Significant *p*-values (<0.05, highlighted), as indicated by Pearson’s chi-square test of independence, suggest that the two responses are related to each other.

	Lame	Hairless	Wounds	Swelling	Discharge	Cough	Listless
Number of cattle affected by injury or disease	0.13	0.02	0.01	0.12	0.02	<0.01	0.03
Number of cattle affected by parasites	<0.01	0.01	0.01	0.01	<0.01	<0.01	<0.01
Availability of first aid and basic medications	<0.01	<0.01	<0.01	0.02	0.01	0.01	0.01
Parasite control programme used	0.03	0.02	0.09	0.12	0.11	0.09	0.27
Vaccination programme used	0.01	0.06	0.01	0.03	0.02	0.05	0.04
Frequency of farmers checking on their cattle	0.32	0.11	0.05	0.01	0.09	0.05	0.02

## Data Availability

The original data presented in the study are openly available in the CSIRO Data Access Portal at https://doi.org/10.25919/nk06-s293.

## Supplementary Materials

The following supporting information can be downloaded at: https://www.mdpi.com/article/10.3390/ani14182666/s1, Survey: Quality of life of beef cattle living in Australian pasture-based systems.
