# Supplementary material for: Influences on Perceived Feasibility of Animal-Based Measures in a Producer-Driven Welfare Benchmarking System
Source: Animals (Basel). 2024 Sep 13;14(18):2666. doi: 10.3390/ani14182666 (PMC11429234; doi:10.3390/ani14182666)
Supplement: Supplementary file 1 [file animals-14-02666-s001.zip › animals-3159611-supplementary.pdf]

Quality of life of beef cattle living in Australian pasture-based systems

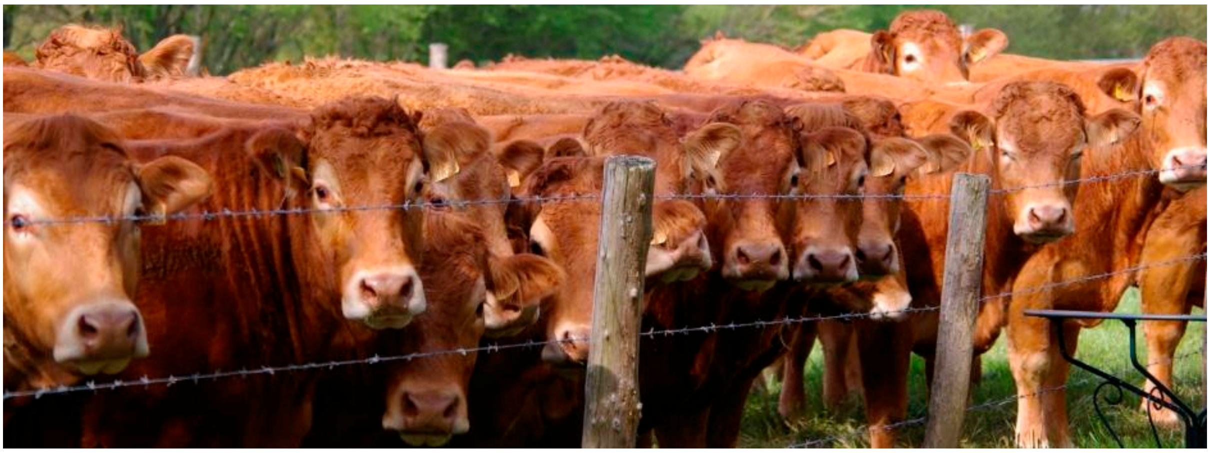

Thank you for taking the time to help with this research project

The survey will be open from the 19th June 2020 and will close at midnight on the 19th July 2020. Participation in this study is completely voluntary and you do not have to take part. You are free to withdraw by stopping at any time. Responses from incomplete surveys will not be included in the final analysis. Aside from giving up your time, there are no foreseeable risks associated with participating in this study. If you choose to complete and submit the survey to us, we take that as your consent to participate in this study. We ask that you only complete the survey once.

We expect to publish and/or present the findings from this study in a variety of forums. All data collected in this study will be anonymous and will be analysed and reported in such a way that responses will not be able to be linked to any individuals. Any data collected as part of this study will be securely stored as per the CSIRO Research Data Management Policy. The de-identified data may also be kept and used for future research on this topic. If you are interested in receiving a copy of the results of this survey when they become available, please email the study leader on the email provided below.

This study has been approved by CSIRO Human Research Ethics Committee in accordance with the National Statement on Ethical Conduct in Human Research (2007) and is funded by CSIRO, NSW DPI and Meat and Livestock Australia. If you have any questions concerning your participation in the study, please contact the study leader.

Alternatively, any concerns or complaints about the conduct of this study can be raised with CSIRO's Executive Manager of Social Responsibility and Ethics.

Demographics – the information you provide here will help us understand who has completed the survey

1. With which gender do you best identify?

☐Female
☐Male
☐Prefer to self-describe:________________
☐Prefer not to say

2. To what age group do you belong?

☐18-24 years old.
☐25-34 years old.
☐35-44 years old.
☐45-54 years old.
☐55-64 years old.
☐65-74 years old.
☐75 and over

3. What is your postcode?

____

Beliefs and understanding

4. Which of the following best describes you?
☐I purchase beef products for my household but don't eat beef
☐I don't eat or purchase beef products
☐I eat beef products

5. How important do you believe it is that animals raised for food production in Australia have a good quality of life?

☐Not important
☐Slightly important
☐Moderately important
☐Important
☐Very important

The following questions relate to pasture-based production systems for beef cattle. Pasture-based systems are those in which cattle are grazed in open paddocks on a predominantly pasture diet.

6. What level of interaction with pasture-based beef farms have you had in the past 10 years?
   ☐I have never visited a pasture-based beef farm
   ☐I have visited a pasture-based beef farm
   ☐I have studied or worked in a role within or related to the beef industry, but I haven't owned or worked on a pasture-based beef cattle farm in Australia
   ☐I have owned or worked on a pasture-based beef cattle farm in Australia
   ☐Other____

7. How would you rate your knowledge of pasture-based beef cattle production systems?

   ☐Non-existent
   ☐Poor
   ☐Fair
   ☐Good
   ☐Excellent

8. For beef cattle living in Australian pasture-based systems, do you believe their overall quality of life is…

   ☐Very poor
   ☐Poor
   ☐Acceptable
   ☐Good
   ☐Very good
   ☐I don't know

Measuring quality of life in beef cattle

For the next set of questions, imagine you have been asked to inspect a pasture-based beef farm and to decide if you think the cattle there have a good quality of life. What do you think would be the **MOST** important things to check on that farm to prove that the cattle have a good quality of life? Try to consider what you would most want to know about regardless of whether you think it would be easy or practical to measure. You don't need to have had experience on a beef farm or be knowledgeable about pasture-based beef cattle production to answer these questions; we are just interested in your ideas.

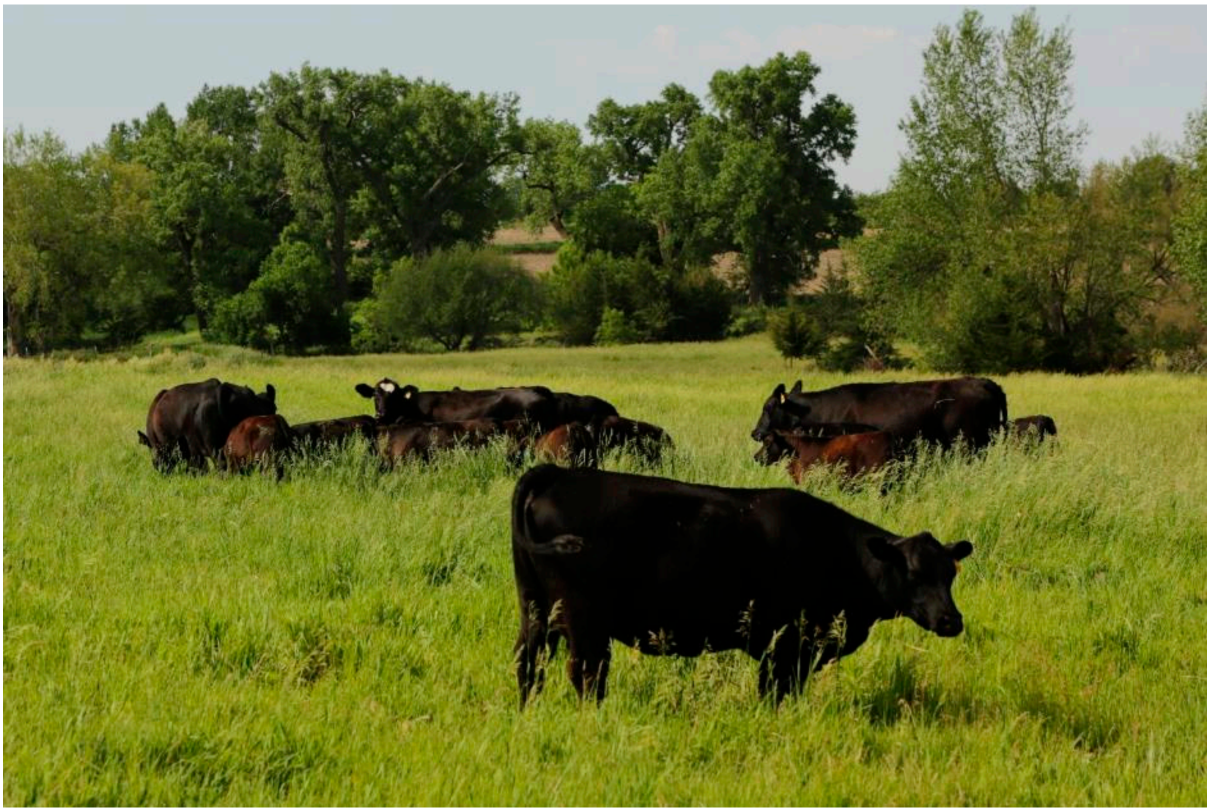

9. Please select the measures relating to **animal health** you think should be checked to prove that cattle have a good quality of life (tick all that apply).

☐Availability of first aid and basic medications to treat sick or injured cattle when needed

☐Number of cattle affected by injury or disease

☐Number of cattle affected by parasites

☐Number of cattle who have died

☐Number of cattle who have calving difficulties

☐Number of cattle killed or injured by predators

☐The methods of euthanasia used

☐Vaccination programme used

☐Parasite control programme used

☐How farmers dispose of dead cattle

☐Other____

☐None of the above need to be checked to prove that cattle have a good quality of life

☐I don't know

10. Please select the measures relating to **the farm environment** you think should be checked to prove that cattle have a good quality of life (tick all that apply).

☐How many cattle have escaped their paddock in the past year

☐How often cattle are exposed to extremely hot or cold weather

☐How much shade cattle have access to in hot weather

☐How much shelter cattle have access to in cold weather

☐The type of fencing used on the farm

☐How often farmers check the fences for damage

☐Other____

☐None of the above need to be checked to prove that cattle have a good quality of life

☐I don't know

11. Please select the measures relating to **food and water** you think should be checked to prove that cattle have a good quality of life (tick all that apply).

☐How often cattle have access to water per day

☐The source of the water they drink (i.e. dam, creek, trough)

☐The quality of the water

☐The temperature of the water

☐The distance they need to walk to get water

☐How often water sources are checked by the farmer

☐The amount of pasture available

☐The number of cattle that are underweight

☐The number of cattle that are overweight

☐The use of additional feeds such as grain, hay or pellets to supplement the pasture diet

☐The variety of plant species in the diet

☐The number of cattle with digestive disorders

☐Other____

☐None of the above need to be checked to prove that cattle have a good quality of life

☐I don't know

12. Please select the measures relating to **farm management** you think should be checked to prove that cattle have a good quality of life (tick all that apply).

☐Method of separating (weaning) calves from their mothers (e.g. yard weaning, fence-line weaning etc.)

☐Ability of farmers to provide for cattle in the event of an emergency (i.e. flood, fire, drought)

☐Frequency that farmers check on their cattle

☐How often cattle are kept on their own without being able to see other cattle

☐How often groups of cattle who are not familiar with each other are mixed together

☐Whether artificial breeding procedures such as artificial insemination or embryo transfer are conducted

☐Whether breeding animals are selected to reduce the risk of health issues in the herd

☐Other____

☐None of the above need to be checked to prove that cattle have a good quality of life

☐I don’t know

13. Please select the measures relating to **handling cattle** you think should be checked to prove that cattle have a good quality of life (tick all that apply).

☐Presence of hazards that may cause animals to trip or fall in the handling facilities (i.e. yards and crush)

☐Presence of sharp or protruding hazards in the handling facilities (i.e. yards and crush)

☐Running the cattle through the handling facilities when the yards are muddy or dusty

☐How long cattle are kept in the handling facilities (i.e. yards) without access to food or water

☐How often cattle are mustered to move them from one paddock to another or to the handling facilities (i.e. yards)

☐The method used to move cattle (e.g. horseback, dogs, motorbike, helicopter, on foot)

☐The aids used to move cattle (e.g. stockwhip, voice, electric prodder, plastic pipe)

☐How often the animals are hit with the aids used to move cattle

☐The speed at which cattle are made to move during mustering

☐How many cattle show signs of overheating or exhaustion after mustering

☐The weather conditions when cattle are mustered

☐How often cattle are mishandled while in the handling facilities (e.g. falls down, attempts to escape, gets stuck, mis-caught in the restraining mechanism of the crush)

☐Other____

☐None of the above need to be checked to prove that cattle have a good quality of life

☐I don’t know

14. Please select the measures relating to **the behaviour of cattle and their handlers (stockpeople)** you think should be checked to prove that cattle have a good quality of life (tick all that apply).

☐How cattle seem after being handled by people (e.g. calm versus fearful)

☐How cattle behave towards new things (e.g. curious versus frightened)

☐How much training the stockpeople have had in handling cattle

☐The attitude of stockpeople towards cattle

☐Whether cattle have been trained to get used to being handled

☐The temperament of the cattle

☐How much time cattle spend resting/lying down

☐Other____

☐None of the above need to be checked to prove that cattle have a good quality of life

☐I don't know

15. Please select the measures relating to **routine husbandry** you think should be checked to prove that cattle have a good quality of life (tick all that apply).

☐Types of routine husbandry conducted on the farm (e.g. castration, branding, dehorning, ear tagging)

☐The age of the cattle on which routine husbandry is performed (e.g. castration, branding, dehorning, ear tagging)

☐How many cattle had complications from a routine husbandry procedure (e.g. infection, excessive bleeding, etc.)

☐Use of pain relief medications to minimise the pain during husbandry procedures (e.g. castration, dehorning, etc.)

☐How often the equipment for routine husbandry is cleaned and disinfected

☐Other____

☐None of the above need to be checked to prove that cattle have a good quality of life

☐I don't know

16. Are there any measures not covered by the above subheadings that you believe should also be checked to prove that cattle have a good quality of life?

____

____

For those who did not select "I have owned or worked on a pasture-based beef cattle farm in Australia" in question 6:

The survey is now complete. Thank you for taking the time to share your thoughts with us.

For those who selected "I have owned or worked on a pasture-based beef cattle farm in Australia" in question 6:

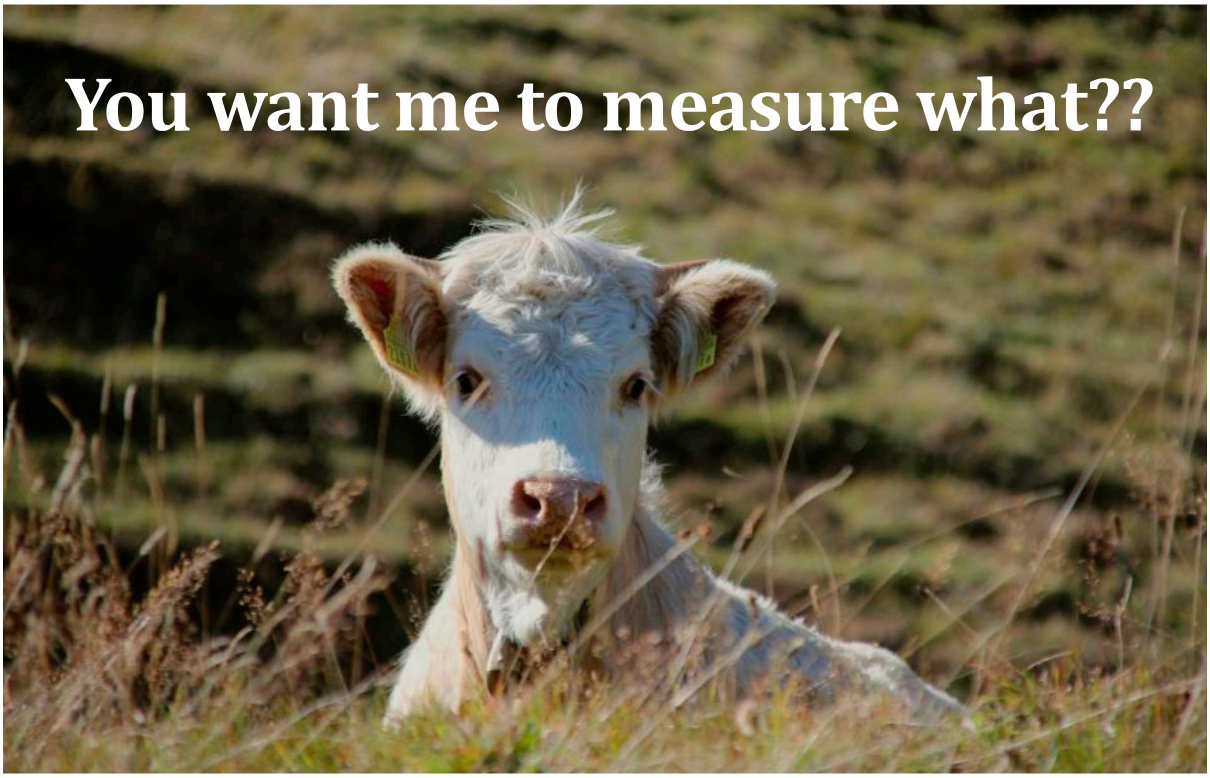

Thank you for sharing your thoughts with us on what you think would be the most important things to check on a farm to prove that the cattle have a good quality of life.

As a person with experience owning or working on a pasture-based beef cattle property in the last 10 years, we would like to ask you a few additional questions on how feasible you think it would be to actually measure some of these things on farm. Many potential measures already exist, however we need to make sure that **producers like you are able to collect these measures** if they are to be included in a benchmarking tool. We are interested in whether you could collect these measures on the property you own or work on and if not, the reasons that would prevent you from doing so. This information will help us determine **which measures make the final cut** in the benchmarking tool. If you choose to stop now we can still use the response you have provided so far.

The additional questions will only take around 10 minutes to complete, so please, grab another cuppa and share your farming expertise with us.

We would first like to learn a little more about the enterprise you own or work on and what would encourage use of a benchmarking tool to assess the quality of life of beef cattle.

1. Approximately how many head of cattle do you have on your property(s) or on the property(s) you work on?

   a. Total herd size = ________
   b. Largest mob size = ________

2. What is the total area of the property/properties? _______________ ha/ac

3. What factors do you think would encourage use a benchmarking tool to assess the quality of life of beef cattle (select all that apply)?

   ☐A price premium at market
   ☐Access to niche markets
   ☐Helping to preserve the right to farm by meeting consumer expectations around quality of life
   ☐Being able to see what other farms are doing to improve quality of life in their cattle
   ☐Being able to see where the property sits compared with my peers in terms of the quality of life provided to the cattle
   ☐Only if it was made compulsory
   ☐Other (please specify)______________________________

Body condition scoring is being considered as one of the potential measures that may be included in a tool to benchmark quality of life in beef cattle at pasture.

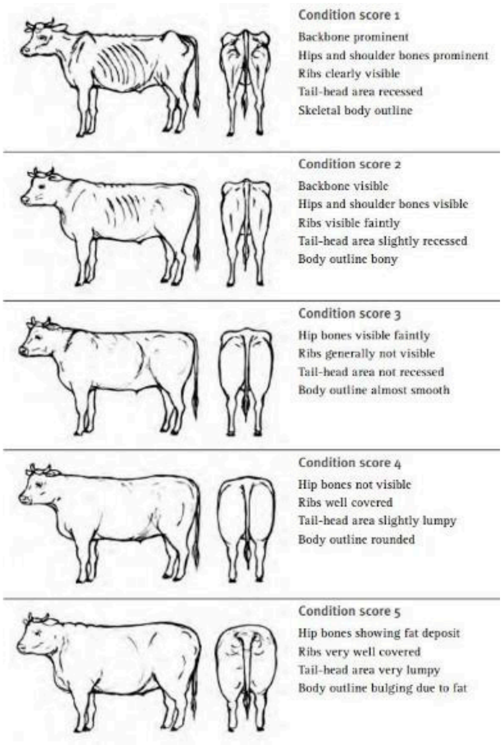

4. How feasible would it be for you to <u>collect and record</u> **body condition scores** for all of the cattle on the property(s)?

**Frequency**	I already collect it	Highly feasible	Feasible	Unfeasible	Highly unfeasible
**Once a year**					
**Multiple times a year**					

a. * asked if selects unfeasible or highly unfeasible to once a year frequency.
You indicated it wouldn't be feasible for you to collect at least one **body condition score** a year on <u>all</u> of the cattle. Would it be feasible for you to collect **body condition scores** for <u>some</u> of the cattle?

☐No

☐Yes, if so, on what percentage of the herd do you think you could collect **body condition scores**? ________ %

b. * asked if selects no to part a
What would be the main reason(s) for you not being able to collect this information (select all that apply)?

☐Time required

☐Additional personnel required

☐Causes additional stress to the cattle

☐Other (please specify)_______________________________

c. * asked if selects feasible or already collects it in first part or Yes in part a.
You indicated that it would be feasible for you to collect at least one **body condition score** on the cattle each year. Please list when you already do or would collect **body condition scores** (e.g. calving, at purchase, in spring etc.)

____

____

Body weight is being considered as one of the potential measures that may be included in a tool to benchmark quality of life in beef cattle at pasture.

5. How feasible would it be for you to collect and record **body weights** for all of the cattle on the property(s)?

**Frequency**	I already collect it	Highly feasible	Feasible	Unfeasible	Highly unfeasible
**Once a year**					
**Multiple times a year**					

a. * asked if selects unfeasible or highly unfeasible to once a year frequency.
You indicated it wouldn't be feasible for you to collect at least one **body weight** a year for all of the cattle. Would it be feasible for you to collect **body weights** for some of the cattle?

☐No

☐Yes, if so, on what percentage of the herd do you think you could collect **body weight**? _______ %

b. * asked if selects no to part a
What would be the main reason(s) for you not being able to collect this information (select all that apply)?

☐Time required

☐Additional personnel required

☐Causes additional stress to the cattle

☐I don't have access to weigh scales

☐Other (please specify)________________________________

c. * asked if selects feasible or already collects it in first part or Yes in part a.
You indicated that it would be feasible for you to collect at least one **body weight** on the cattle. Please list when you already do or would collect **body weight** (e.g. calving, at purchase, in spring etc.)

____

____

Temperament is being considered as one of the potential measures that may be included in a tool to benchmark quality of life in beef cattle at pasture. Temperament can be scored using a combination of two measures. Crush exit score is the speed at which cattle move when released from the crush (i.e. walk, trot, run or jump). Crush score is the level of agitation cattle display while res trained in a crush.

Crush score	Description
**1**	Calm standing still, head mostly still, and slow, calm movements
**2**	Slightly restless looking around more quickly, moving feet, and shifting weight
**3**	Restless moving backward and forward, and some slight movement of crush
**4**	Nervous continuous vigorous movement backward and forward, snorting, and some movement of crush
**5**	Very nervous violent movements, rearing, and attempting to jump out

6. How feasible would it be for you to <u>collect and record</u> **temperament scores** for all of the cattle on the property(s)?

**Frequency**	I already collect it	Highly feasible	Feasible	Unfeasible	Highly unfeasible
**Once per animal while they are on the property**					
**Multiple times per animal while they are on the property**					

a. * asked if selects unfeasible or highly unfeasible to at least once frequency.
You indicated it wouldn't be feasible for you to collect **temperament scores** at least once for all of the cattle. Would it be feasible for you to collect **temperament scores** at least once while on the property for <u>some</u> of the cattle?

☐No

☐Yes, if so, on what percentage of the herd do you think you could collect **temperament scores**? ________ %

b. * asked if selects no to part a
What would be the main reason(s) for you not being able to collect this information? (select all that apply)

☐Time required

☐Additional personnel required

☐Causes additional stress to the cattle

☐Other (please specify)________________________________

The number of cattle with **health issues** is being considered as one of the potential measures that may be included in a tool to benchmark quality of life in beef cattle at pasture.

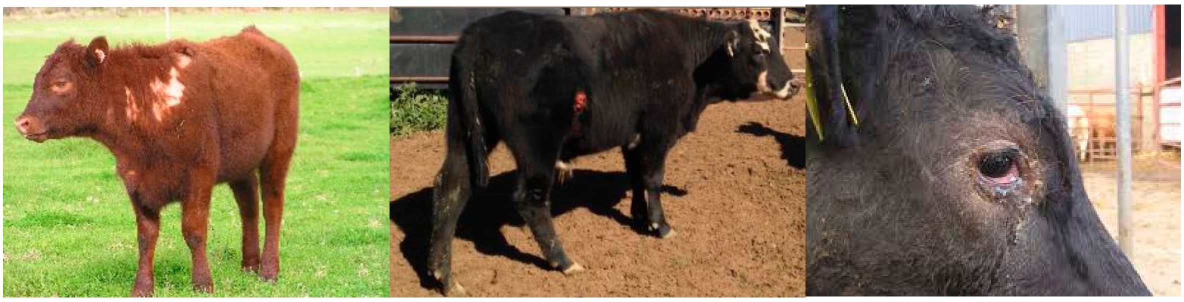

7. How feasible would it be for you to collect and record the number of cattle with the following **health issues** when inspecting them?

Issue	I already collect it	Highly feasible	Feasible	Unfeasible	Highly unfeasible
**Lameness**					
**Hairless patches**					
**Wounds, scabs and/or fresh scars bigger than a 50c coin**					
**Swellings bigger than a golf ball**					
**Eye or nasal discharge**					
**Coughing or laboured breathing**					
**listless or ill looking**					

* asked if selects unfeasible or highly unfeasible to at least one issue.

d. You indicated it wouldn't be feasible for you to record the number of cattle with **health issues** for at least one of the health issues listed above. What would be the main reason(s) for you not being able to collect this information? (select all that apply)

☐Time required

☐Additional personnel required

☐I can't get close enough to the cattle to see these things

☐Other (please specify)________________________________

The number of cows with **calving difficulties** is being considered as one of the potential measures that may be included in a tool to benchmark quality of life in beef cattle at pasture.

Score	Category	Description
1	Unassisted	Cow calved unassisted/no difficulty
2	Easy pull	One person without mechanical assistance
3	Hard pull	Two people without mechanical assistance<br>Once person with mechanical assistance
4	Surgical assistance	Veterinary intervention required
5	Mal presentation	E.g. breech
6	Elective surgery	Surgical removal of the calf before the cow has had a chance to calve

8. How feasible would it be for you to collect and record how many of the cows have **calving difficulties**? (select NA if you do not run/work on a cow-calf operation)

**Issue**	I already collect it	Highly feasible	Feasible	Unfeasible	Highly unfeasible	NA
**Calving difficulties**						

* asked if selects unfeasible or highly unfeasible.

e. You indicated it wouldn't be feasible for you to record the number of **calving difficulties** in the cows. What would be the main reason(s) for you not being able to collect this information? (select all that apply)

☐Time required

☐Additional personnel required

☐I don't check them during calving

☐Causes additional stress to the cattle

☐Other (please specify)________________________________

Signs of exhaustion and heat stress during or following mustering are being considered as potential measures that may be included in a tool to benchmark quality of life in beef cattle at pasture.

**Signs of heat stress and exhaustion**
Open mouth panting and drooling
Muscle tremors in hind legs and shoulders
Unsteady gait
Non-responsiveness
Rapid breathing rate
Head and neck drooping

9. How feasible would it be for you to collect and record the number of cattle showing **signs of exhaustion and heat stress** during mustering or in the first 5 minutes following mustering?

**Time point**	I already collect it	Highly feasible	Feasible	Unfeasible	Highly unfeasible
**Mustering to a new paddock**					
**Mustering to the yards**					

a. *only asked if unfeasible or highly unfeasible to paddock
You indicated it wouldn't be feasible when mustering to a new paddock. What would be the main reason(s) for you not being able to record the number of cattle showing **signs of exhaustion and heat stress**? (select all that apply)

☐Time required

☐Additional personnel required

☐I can't see them during mustering or for the first 5 minutes after mustering

☐Other (please specify)_______________________________

b. *only asked if unfeasible or highly unfeasible to yards
You indicated it wouldn't be feasible when mustering to the yards. What would be the main reason(s) for you not being able to record the number of cattle showing **signs of exhaustion and heat stress**? (select all that apply)

☐Time required

☐Additional personnel required

☐Other (please specify)_______________________________

How the herd seems (herd demeanour) after interacting with people is being considered as one of the potential measures that may be included in a tool to benchmark quality of life in beef cattle at pasture. Observing and scoring herd demeanour may be done by describing what proportion of the herd seem agitated, relaxed, curious, sociable, fearful etc. An example of the way these behaviours may be observed and scored is given below.

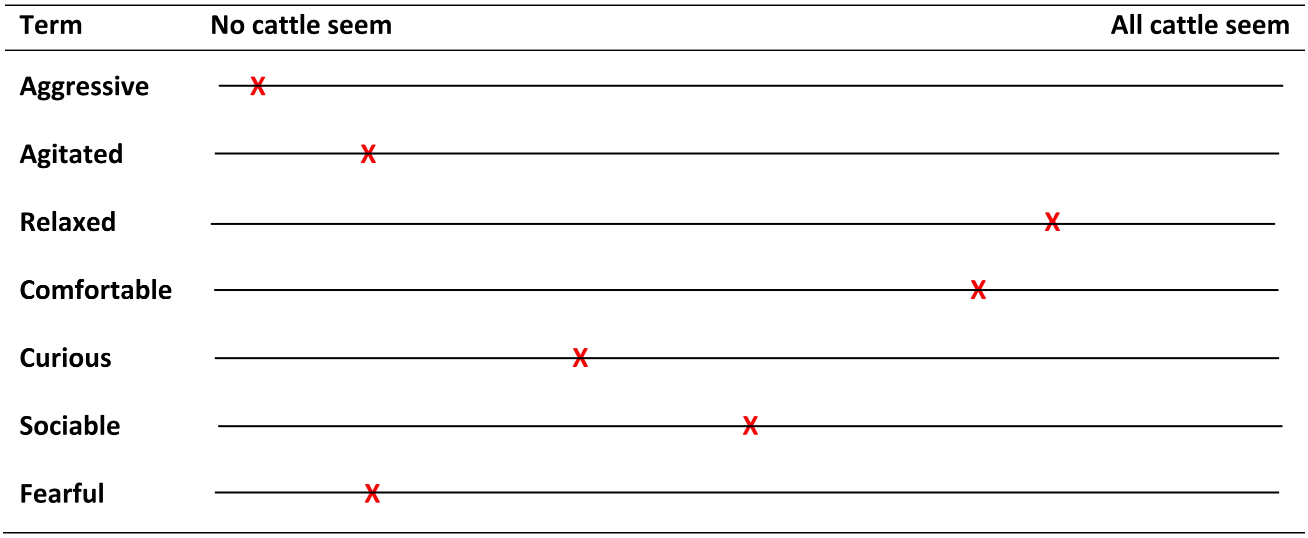

10. How feasible would it be for you to <u>observe and record</u> a **herds demeanour** for a total of 3 minutes in the first 5 minutes after mustering to the yards?

**Time point**	I already collect it	Highly feasible	Feasible	Unfeasible	Highly unfeasible
**Mustering to the yards**					

a. *only asked if unfeasible or very unfeasible
You indicated it wouldn't be feasible for you to record **herd demeanour when mustering to the yards**. What would be the main reason(s) for you not being able to collect this information? (select all that apply)

☐Time required

☐Additional personnel required

☐Other (please specify)________________________________

Herd behaviour and the quality of handling that occurs **during mustering and yarding** are being considered as potential measures that may be included in a tool to benchmark quality of life in beef cattle at pasture.

11. How confident would you be in recording the following scenarios either during or after mustering?

**Scenario**	Confident to record accurately	Confident to record an estimate	It is too hard to see the cattle during mustering to record this	It would take too long to record this	It is too hard to keep track of the details while mustering	Other (please specify below)	I don't use dogs to muster
**How often your dog(s) bit the cattle during mustering**							
**The average speed cattle travelled during mustering (e.g. walk, trot, run)**							
**How often individuals or groups of cattle broke away from your control during mustering**							

If you have any comments or selected "Other" for one of more of the above scenarios, please give more information here:

____

____

12. How confident would you be in recording the following things either during or after handling your cattle in the yards?

Scenario	Confident to record accurately	Confident to record an estimate	It is too hard to see the cattle during yarding to record this	It would take too long to record this	It is too hard to keep track of the details during yarding	Other (please specify below)
**The percentage of cattle requiring additional force to move (e.g. strong physical contact, electric prod, tail twist etc.)**						
**How many cattle trip or fall in the yards/race/crush**						
**How many cattle turn around or get stuck in the race/crush**						
**How many cattle are mis-caught in the head-bail of the crush (e.g. around head, shoulder, leg etc.)**						
**How many cattle try to escape (e.g. climbing rails, jumping out)**						
**How many cattle vocalise prior to having any procedure done**						

**How many locations in the yards where cattle flow is regularly inhibited (e.g. baulking etc.)**						

If you have any comments or selected “Other” for one of more of the above scenarios, please give more information here:

____

____

The attitude of stockpeople towards cattle is being considered as one of the potential measures that may be included in a tool to benchmark quality of life in beef cattle at pasture. This would be done by asking stockpeople to complete a short questionnaire on the extent to which they agree or disagree with statements such as "I enjoy working with cattle", "Cattle are stubborn and hard to handle", "Cattle feel pain when hit with movement aids" or "Cattle have a good memory" (examples only).

13. Would you have any concerns about completing a stockperson attitude questionnaire yourself?

    ☐Yes – Please provide details________________________________
    ☐No
    ☐I am not involved in handling cattle

14. Would you have any concerns about asking anyone else that handles cattle (e.g. family or other staff) to complete a stockperson attitude questionnaire?

    ☐Yes – Please provide details________________________________
    ☐No
    ☐No one else handles the cattle

The survey is now complete. Thank you for taking the time to share your thoughts with us.
